# The XBI BioLab for life science experiments at the European XFEL

**DOI:** 10.1107/S1600576720013989

**Published:** 2021-02-01

**Authors:** Huijong Han, Ekaterina Round, Robin Schubert, Yasmin Gül, Jana Makroczyová, Domingo Meza, Philipp Heuser, Martin Aepfelbacher, Imrich Barák, Christian Betzel, Petra Fromme, Inari Kursula, Poul Nissen, Elena Tereschenko, Joachim Schulz, Charlotte Uetrecht, Jozef Ulicný, Matthias Wilmanns, Janos Hajdu, Victor S. Lamzin, Kristina Lorenzen

**Affiliations:** a European XFEL GmbH, Holzkoppel 4, 22869 Schenefeld, Germany; b Biocenter Oulu and Faculty of Biochemistry and Molecular Medicine, University of Oulu, Aapistie 7, 90220 Oulu, Finland; c European Molecular Biology Laboratory, Notkestrasse 85, 22607 Hamburg, Germany; d Institute of Biochemistry and Molecular Biology, Laboratory for Structural Biology of Infection and Inflammation, University of Hamburg, c/o DESY, Building 22a, Notkestrasse 85, 22603 Hamburg, Germany; e The Hamburg Centre for Ultrafast Imaging (CUI), Luruper Chaussee 149, 22761 Hamburg, Germany; f Institute of Molecular Biology, Slovak Academy of Sciences, Dúbravská cesta 21, 845 51 Bratislava, Slovak Republic; g Biodesign Center for Applied Structural Discovery and School of Molecular Sciences, Arizona State University, Tempe, AZ, USA; h Institute of Medical Microbiology, Virology and Hygiene, University Medical Center Hamburg Eppendorf, Martinistrasse 52, 20246 Hamburg, Germany; i Department of Biomedicine, University of Bergen, Jonas Lies vei 91, 5009 Bergen, Norway; j DANDRITE, Nordic EMBL Partnership for Molecular Medicine, Aarhus University, Department of Molecular Biology and Genetics, Gustav Wieds Vej 10C, DK – 8000 Aarhus C, Denmark; k Institute of Crystallography, Russian Academy of Sciences, 59 Leninsky prospekt, Moscow, 117333, Russian Federation; l Heinrich Pette Institute, Leibniz Institute for Experimental Virology, Martinistrasse 52, 20251 Hamburg, Germany; m Department of Biophysics, Institute of Physics, Faculty of Science, P. J. Šafárik University, Jesenná 5, 04154 Košice, Slovak Republic; n The European Extreme Light Infrastructure, Institute of Physics, Academy of Sciences of the Czech Republic, Za Radnici 835, 25241 Dolní Břežany, Czech Republic; o Laboratory of Molecular Biophysics, Department of Cell and Molecular Biology, Uppsala University, Husargatan 3 (Box 596), SE-751 24 Uppsala, Sweden

**Keywords:** free-electron lasers (XFELs), European XFEL (EuXFEL), XBI Laboratory, serial femtosecond crystallography (SFX), single-particle imaging (SPI), coherent diffractive imaging (CDI), time-resolved experiments, structural biology, sample preparation and characterization

## Abstract

The science of X-ray free-electron lasers (XFELs) critically depends on the performance of the X-ray laser and on the quality of the samples placed into the X-ray beam. The stability of biological samples is limited and key biomolecular transformations occur on short timescales. Experiments in biology require a support laboratory in the immediate vicinity of the beamlines. The XBI BioLab of the European XFEL (XBI denotes XFEL Biology Infrastructure) is an integrated user facility connected to the beamlines for supporting a wide range of biological experiments. The laboratory was financed and built by a collaboration between the European XFEL and the XBI User Consortium, whose members come from Finland, Germany, the Slovak Republic, Sweden and the USA, with observers from Denmark and the Russian Federation. Arranged around a central wet laboratory, the XBI BioLab provides facilities for sample preparation and scoring, laboratories for growing prokaryotic and eukaryotic cells, a Bio Safety Level 2 laboratory, sample purification and characterization facilities, a crystallization laboratory, an anaerobic laboratory, an aerosol laboratory, a vacuum laboratory for injector tests, and laboratories for optical microscopy, atomic force microscopy and electron microscopy. Here, an overview of the XBI facility is given and some of the results of the first user experiments are highlighted.

## Abstract 2

An overview of a unique life science facility at the European XFEL is provided, and its importance in sample preparation, characterization and analysis prior to measurements at the different European XFEL instruments is discussed. The capabilities that the facility can provide for the research community are highlighted, alongside examples of recent successful studies.

## 1. Introduction   

Ultra-short and extremely bright coherent X-ray pulses from X-ray free-electron lasers (XFELs) open up unprecedented research opportunities in physics, chemistry and biology. Intense femtosecond X-ray pulses can outrun key damage processes in the sample and allow researchers to obtain interpretable diffraction patterns beyond conventional damage limits through ‘diffraction before destruction’ (Chapman *et al.*, 2006 ▸, 2011 ▸; Neutze *et al.*, 2000 ▸; Wiedorn *et al.*, 2018 ▸). The precise timing and the synchronization of the X-ray pulses with other photon pulses permit studies on the dynamics of molecular systems with high temporal and spatial resolution (Barends *et al.*, 2015 ▸; Nogly *et al.*, 2018 ▸; Pande *et al.*, 2016 ▸; Pandey *et al.*, 2020 ▸; Tenboer *et al.*, 2014 ▸; Woodhouse *et al.*, 2020 ▸).

Biological processes occur over multiple orders of magnitude in time and over multiple levels of structural organization and from atoms to molecules and cells and beyond. For every isolated component of an organism, appropriate conditions need to be met for keeping it stable, active and structurally intact outside of its natural environment. XFEL studies may include capturing femtosecond intermediates in photochemical reactions and experiments on viral infections or on programmed cell death. As a consequence, the support laboratory has to satisfy a significantly wider range of requirements than most remote home laboratories, to allow rapid sample delivery to the XFEL instruments.

### 1.1. The XFEL Biology Infrastructure   

In February 2011, the Council of the European XFEL (EuXFEL) decided to consider contributions by user consortia to the construction of end-stations and various other infrastructures for the operation of the EuXFEL. In response to this call, the XBI User Consortium was initiated by Janos Hajdu and Victor Lamzin. The aim of the User Consortium was to create an ‘XFEL Biology Infrastructure’ (hence the acronym XBI) to maximize the efficient use of the EuXFEL and to minimize the likelihood of experiment failure. Scientists from the European Molecular Biology Laboratory (EMBL, Hamburg), Finland (University of Oulu), Germany (University Hospital Eppendorf, University of Hamburg), the Slovak Republic (Ministry of Education, Science Research and Sport of the Slovak Republic), Sweden (Uppsala University) and the USA (Arizona State University) joined forces with the European XFEL to establish the new biology infrastructure, with additional input by observers from Denmark and the Russian Federation. The XBI User Consortium has contributed finances, instrumentation and personnel to the design, construction, commissioning and operation of the XBI BioLab, which opened its doors to the international research community in the summer of 2017.

**Figure 1:**
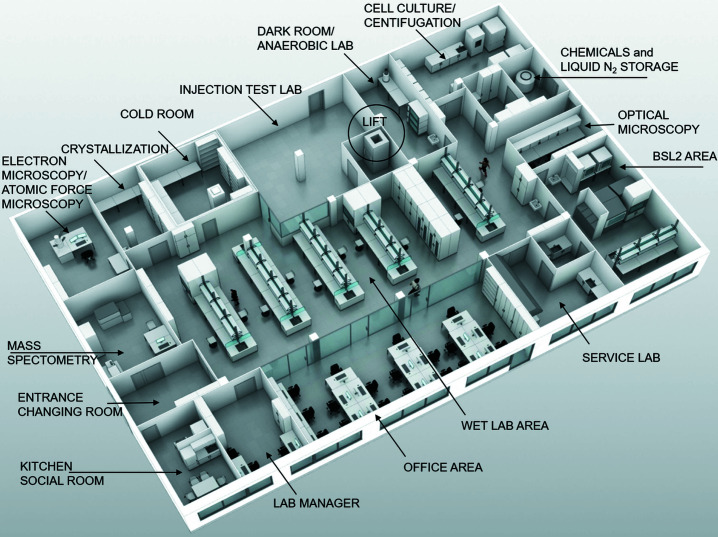
The layout of the XBI BioLab with the main instrument areas marked. The base-line facility was built according to the state of the art in 2016. It includes a local low-vacuum system and gas supply as well as safety-relevant features such as fume hoods, safety workbenches and oxygen monitors (where necessary). The laboratory also has an office area for users and a small kitchenette for food and rest. The XBI BioLab is right above the SPB/SFX instrument of the EuXFEL and it is directly connected to the experiment areas via a sample lift. Graphics by Florian Ott, Hamburg.

The overall layout of the large XBI laboratory is shown in Fig. 1 ▸. The laboratory is located in the headquarters of the EuXFEL in Schenefeld, right above the X-ray instruments of the free-electron laser. The XBI permits experiments on living cells, cell organelles, single virus particles, macromolecular complexes, single biomolecules, and nano- and microcrystals, as well as other materials. The laboratory is directly connected to the instruments of the EuXFEL via a sample lift. The XBI facility operates as an integral component of the EuXFEL and provides support to users of various scientific instruments as well as to a wider research community. The needs and requirements of the EuXFEL users from the life science community were central in the design of the facility. Functional rooms with specialized equipment are grouped around a central area housing the general laboratory infrastructure (Fig. 1 ▸). This allows multiple user groups to work comfortably and independently at the same time. Users are able to start sample production from genetic material if needed. Large-scale bacterial or eukaryotic cell cultures can be prepared using incubators or a fermenter. Various expression systems and cells are available. Special illumination conditions can be selected for photosynthetic microorganisms or light-sensitive samples. Samples can be handled under controlled parameters, such as temperature, humidity and gas environment, with quality control at each point of sample preparation. The XBI laboratory is equipped with an anaerobic glove box for oxygen-free and tailored atmospheric composition, being located in a room fitted with variable light conditions, so even highly reactive samples sensitive to oxygen and light can be prepared. Two chromatography systems are available in the laboratory, one in the cold room and the other at room temperature, along with a wide range of columns for protein purification. The purified samples can be characterized in terms of quality and activity. Support with up-scaling of protein production ensures that enough material can be obtained for testing, for optimization and eventually for the experiment at the instruments. The XBI laboratory has a special focus on the biophysical characterization of samples, as outlined in detail below.

### 1.2. Experiments in structural biology   

The European X-ray Free-Electron Laser is an exceptionally bright X-ray source and permits studies on both crystalline and noncrystalline samples. As of today, the most common experimental approaches are room-temperature serial femtosecond X-ray crystallography (SFX) (Boutet *et al.*, 2012 ▸; Chapman *et al.*, 2011 ▸; Martin-Garcia *et al.*, 2016 ▸; Mills *et al.*, 2020 ▸; Schlichting, 2015 ▸) and single-particle imaging (SPI) (Ekeberg *et al.*, 2016 ▸; Neutze *et al.*, 2000 ▸; van der Schot *et al.*, 2015 ▸; Seibert *et al.*, 2011 ▸; Sobolev *et al.*, 2020 ▸). Both techniques can be combined with simultaneous spectroscopic measurements. Currently, the European XFEL is the only facility worldwide to enable experiments at megahertz repetition rates. This unprecedented data collection rate together with submicrometre beam focusing (Bean *et al.*, 2016 ▸; Mancuso *et al.*, 2019 ▸) and the application of either cage-based reaction initiation (Bourgeois & Weik, 2009 ▸; Duke *et al.*, 1992 ▸; Schlichting *et al.*, 1990 ▸; Stoddard *et al.*, 1998 ▸; Tosha *et al.*, 2017 ▸) or mix-on-demand sample delivery (Beyerlein *et al.*, 2017 ▸; Ishigami *et al.*, 2019 ▸; Knoška *et al.*, 2020 ▸; Kupitz *et al.*, 2017 ▸; Oberthuer *et al.*, 2017 ▸; Schmidt, 2013 ▸; Stagno *et al.*, 2017 ▸; Wang *et al.*, 2014 ▸) will allow researchers to trigger and film chemical reactions in rapid time-resolved experiments to obtain three-dimensional movies of the nanoworld.

Several end-stations of the EuXFEL are available for life science applications. The main station is the SPB/SFX (Single Particles, Clusters and Biomolecules & Serial Femtosecond Crystallography) instrument (Mancuso *et al.*, 2019 ▸; Mills *et al.*, 2020 ▸) located on the SASE1 (self-amplified spontaneous emission) undulator line. This instrument can be configured for optimized measurements in serial-femtosecond crystallography or in single-particle imaging. Other beamlines and instruments are also available for studies in the life sciences, including the Femtosecond X-ray Experiments (FXE) instrument (Bressler *et al.*, 2012 ▸) on the SASE1 undulator for studies of enzyme kinetics to capture transient intermediates, the Material Imaging and Dynamics (MID) instrument (Madsen *et al.*, 2013 ▸) on the SASE2 undulator for limited-flux whole-cell time-resolved tomography studies, and the brightest instrument of the EuXFEL, the Spectroscopy and Coherent Scattering (SCS) station (Scherz *et al.*, 2013 ▸), as well as the Small Quantum Systems (SQS) instrument, both on the SASE3 undulator section.

### 1.3. Support for experiments   

**Figure 2:**
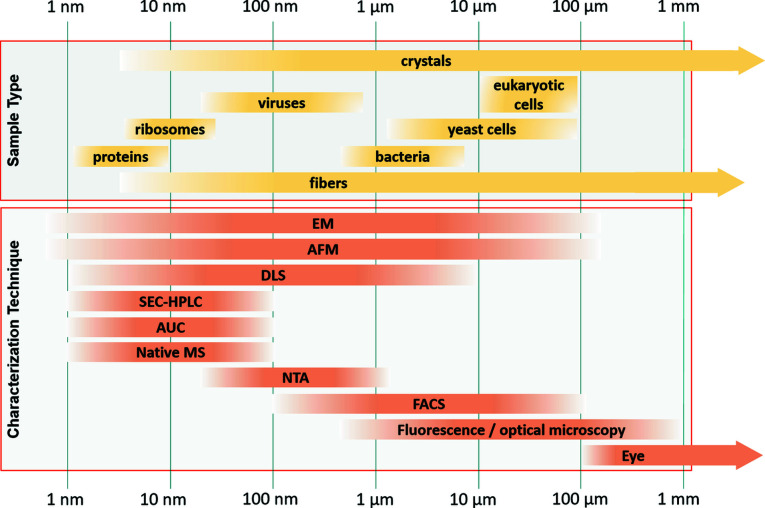
Overview of XBI characterization techniques for analysis of nano/microcrystals and particles of different types. EM – electron microscopy, AFM – atomic force microscopy, DLS – dynamic light scattering, SEC-HPLC – size-exclusion chromatography, high-performance liquid chromatography, AUC – analytical ultracentrifugation, native MS – native mass spectrometry, NTA – nanoparticle tracking analysis, FACS – fluorescence-activated cell sorting.

X-ray free-electron lasers are in high demand worldwide and beam time access is highly competitive. The availability of a large sample preparation laboratory can help secure success of the experiments at the beamlines. The XBI BioLab has a wide scope to enable work on a broad range of biological specimens from cells to single macromolecules (Fig. 2 ▸). The XBI support team provides assistance in the production, preparation, optimization and characterization of different types of samples. The laboratory offers test chambers and diagnostic instruments to mimic XFEL experiments and to assure successful sample injection. Users are provided with an opportunity to identify and address sample-related problems ahead of beam time.

### 1.4. Sample preparation and characterization   

*Prokaryotic cell systems*. The most commonly used technique to produce sample proteins is expression of recombinant genes in bacterial cells like *Escherichia coli*. The XBI BioLab offers a set of instruments for such purposes, including a polymerase chain reaction machine, a gel documentation system, incubators and shakers, as well as a fermenter for preparing large-scale fed-batch cell cultures for work on organisms at biological safety level 1 (BSL1). There is an option of growing photosynthetic bacteria as well.

*Eukaryotic cell systems*. Microbiological safety benches are located in the area classified for work at biological safety level 2 (BSL2) and allow culture of insect cells or mammalian cells. Incubators and shakers are available along with a cell sorter.

*Protein purification*. Cells can be lysed by a sonicator or an emulsifier, and proteins can be purified using two available chromatography systems with a variety of different columns and an ultracentrifuge with several rotors covering a broad range of experiments.

*Facilities for handling highly sensitive samples*. The XBI BioLab has a cold room, a ‘dark laboratory’ where illumination conditions can be adjusted for work on light-sensitive samples, and an anaerobic laboratory area with a glove box for experiments under a specific atmosphere, minimized oxygen concentration and controlled humidity.

*Dynamic light scattering and nanoparticle tracking analysis*. After protein purification, the oligomeric state and homogeneity of the sample solution can be analysed by dynamic light scattering (DLS) or nanoparticle tracking analysis (NTA). Both dynamic light scattering and nanoparticle tracking analysis measure the Brownian motion of nanoparticles whose speed of motion, or diffusion constant, is related to particle size. Both methods provide information about particle size and NTA allows determination of the particle concentration as well. For DLS measurements, the XBI laboratory offers a cuvette instrument as well as a plate reader, both equipped with an infrared laser (785 nm) and an additional laser with 660 nm excitation wavelength in the plate reader, allowing measurements on samples absorbing light at optical wavelengths. The required sample volume for DLS is between 0.1 and 4 µl, and the measuring range of hydro­dynamic radii is from 0.25 to 2500 nm. The sample volume for NTA measurements is about 100 times higher, but the sample concentration is about 100 times more dilute compared with DLS. The size range of particles in NTA is between 20 and 1000 nm in diameter.

*Analytical ultracentrifugation*. Before crystallization the stoichiometry and stability of the sample can be evaluated by analytical ultracentrifugation (AUC). AUC measures the radial concentration gradient of the sample using absorbance and interference during sedimentation, and characterizes the Svedberg constant, mass, size, anisotropy and association properties of the sample.

*Spectroscopy*. A UV–vis spectrophotometer and a nanodrop are available for concentration determination and absorption spectra in droplets or using cuvettes, while a plate reader allows performing activity assays based on absorption, fluorescence or luminescence.

*Light microscopy*. For visual inspection of crystallization or cell culture plates, several stereomicroscopes and fluorescence microscopes are available in the XBI Biolab, the latter both inverted and upright systems with a set of exchangeable objectives, filters and cameras.

*Atomic force microscopy and transmission electron microscopy*. The XBI laboratory offers complementary possibilities to image the sample using atomic force microscopy (AFM) and transmission electron microscopy (TEM). The available bio-AFM allows imaging of immobilized particles in aqueous and dry environments. AFM images provide topographical information on the sample with sub-nanometre resolution in **Z** and a few nanometre resolution in the **XY** directions, depending on the cantilever. Furthermore, AFM can be used to determine the Young modulus of the sample, providing information on the particle stiffness (Marchetti *et al.*, 2016 ▸). The TEM instrument provides high resolution for single-molecule imaging and can be used to identify protein nanocrystals, too small to be seen using light microscopy. Here, a widely applied method is to increase the contrast of the biomolecules by staining with uranyl acetate (negative staining). Although the grain size of the uranyl acetate limits the obtainable resolution to about 1 nm, negative staining offers a fast and efficient tool to characterize the sample by TEM. The polydispersity of the sample can be judged and the integrity of the particles to be imaged can be assessed. As the penetration depth of electrons is limited to a few hundred nanometres, larger samples such as cells or larger protein crystals can be embedded in epoxy resin for sectioning using the available (cryo) ultramicrotome. A second method for TEM is imaging samples under cryo conditions as a potential way to characterize conformational heterogeneity and to gain additional valuable information for binning and reconstruction of SPI data. The software *serialEM* (Mastronarde, 2005 ▸) is installed at the TEM instrument, for automated low-dose image recording.

*Crystallization robots*. The laboratory offers a liquid handling and crystallization robot for testing crystallization conditions and to optimize crystal size and quality for soluble proteins, as well as for membrane proteins in lipidic cubic phase (LCP). Storage options are available in several incubators with precise temperature control.

*Crystallization plate imaging*. Initial inspection of the crystals can be carried out with high-resolution stereomicroscopes. The XBI laboratory also offers second-order nonlinear imaging of chiral crystals (SONICC), utilizing second harmonic generation (SHG) and ultraviolet two-photon excited fluorescence (UV-TPEF) methods for the identification of small chiral crystals. Only chiral protein crystals produce both SHG and UV-TPEF signals, making it possible to differentiate such microcrystals from the precipitate.

## 2. Samples for serial femtosecond crystallography   

SFX is currently one of the most common methods used at XFELs for structure determination. At the SPB/SFX instrument, 75% of user beam time has been allocated to this type of experiment to date. Owing to the megahertz pulse trains and the femtosecond pulse duration of the EuXFEL, the instrument is highly suited for time-resolved experiments of proteins in action, with the ability to yield structures of short-lived intermediates which cannot be obtained by other methods. The high peak brilliance offers the possibility to obtain diffraction patterns from micrometre and sub-micrometre-sized crystals (Falkner *et al.*, 2005 ▸; Gati *et al.*, 2017 ▸; Orville, 2020 ▸), where the diffusion of ligands into protein crystals is fast enough for timescales relevant to biological reactions (Bar-Even *et al.*, 2011 ▸; Hajdu *et al.*, 2000 ▸; Hajdu, Acharya *et al.*, 1987 ▸; Hajdu, Machin *et al.*, 1987 ▸; Schmidt, 2013 ▸). Homogeneity in crystal size is, therefore, a crucial factor in time-resolved experiments, where important biological and pharmacological reactions can be observed in real time at near-atomic spatial resolution. Temporal resolution is limited by the time for the substrate (in the case of solution mixing) or light (in the case of optical activation) to proceed into the crystal. Uniform photoactivation strongly depends on the penetration depth of the pump pulse. The crystal size should therefore be small enough to permit the excitation of the entire crystal. The actual size of each single crystal is an essential parameter to achieve homogenous reaction initiation with photons and also through diffusion (Grünbein *et al.*, 2020 ▸).

One of the benefits of SFX at XFELs is that, owing to the diffraction-before-destruction principle, it is possible to carry out the measurement in physiological buffer conditions at room temperature, instead of using flash-frozen samples. Flash-freezing preserves the sample in a stable state, but it can cause artefacts and poses challenges for investigation of dynamics. Having the sample in a non-frozen state can lead to degradation, proteolysis, processing, dissolving or growth of oversized crystals, and these must be avoided. Thus, sample preparation in the vicinity of the experiment is crucial, to minimize unwanted degradation processes and other changes of the sample integrity.

At XFELs, each nano- or microcrystal is usually only shot once by a single short X-ray pulse, and through this pulse the crystal is destroyed. A 3D reconstruction is assembled from tens of thousands of 2D diffraction patterns obtained from individual crystals. This means that sample preparation for SFX experiments requires production of large numbers of crystals with homogeneous size distribution and high number density. DLS can be used to investigate the size distribution of nanocrystals of smaller than 5 µm and to observe and follow the crystallization process (Schubert *et al.*, 2015 ▸; de Wijn *et al.*, 2020 ▸). Once an initial hit is identified, optimization screens can be prepared using the liquid-handling system to find conditions producing micro- or nanocrystals with homogeneous sizes at high enough concentration.

### 2.1. Test facility for injecting nano- and microcrystals into the XFEL beam   

Some protein samples cannot be frozen at any step during the isolation and crystallization process, and these proteins need to be freshly produced and purified before the beam time and then crystallized on site. Alternatively, crystals can be shipped and re-crystallized on site or crushed and used as seed stocks for batch crystallization to yield a homogenous crystal suspension. Nano/microcrystals can be delivered to the X-ray beam using a variety of methods, including liquid jets created by gas dynamic virtual nozzles (GDVNs) (DePonte *et al.*, 2008 ▸; Oberthuer *et al.*, 2017 ▸; Stan *et al.*, 2016 ▸). The method of choice is dependent on the requirements and availability of the sample (Cheng, 2020 ▸; Grünbein & Nass Kovacs, 2019 ▸; Martiel *et al.*, 2019 ▸). Heterogeneous mixtures of crystals can clog nozzles and lead to extended down times during beam time. The settling or the viscosity of the crystal slurry can also cause problems even with crystals of uniform size. Testing the injection of nano- and microcrystals in liquid jets prior to experiments is crucial for the success of data collection. Time-resolved experiments at megahertz rates require high-speed injection in order to replenish the sample, and thus GDVN-based liquid jets are usually the favoured choice. The desired jet speed for the high-repetition beam pulses at the European XFEL is over 50 m s^−1^ to mitigate shock waves from the sample explosion and crystal damage (Mills *et al.*, 2020 ▸; Nass, Gorel *et al.*, 2020 ▸; Stan *et al.*, 2016 ▸; Wiedorn *et al.*, 2018 ▸). Although GDVNs are perhaps the most successful injectors at XFELs, they have one critical disadvantage: a very high sample consumption (Coe & Ros, 2018 ▸; Echelmeier *et al.*, 2020 ▸). Most of the crystals are lost without any interaction with the X-ray beam during the ‘dark time’ between X-ray pulses.

When sample availability is limited, low-speed injectors based on high-viscosity extrusion (HVE) techniques can be employed (James *et al.*, 2019 ▸; Liu *et al.*, 2013 ▸; Weierstall *et al.*, 2014 ▸). For membrane protein crystals grown in highly viscous LCP, HVE injection is advantageous, as it allows data collection from microcrystals grown in their ‘native’ lipidic environment (Feld & Frank, 2014 ▸; Standfuss, 2019 ▸). HVE injectors reduce mechanical stress by minimizing crystal manipulation. In addition to the membrane protein, HVE injection can be applied to the protein crystals grown in aqueous solutions. Besides LCP, other chemicals have been identified and characterized as viscous delivery media to embed such crystals (Nam, 2019 ▸), *e.g.* Vaseline (Botha *et al.*, 2015 ▸), agarose (Conrad *et al.*, 2015 ▸) and hyaluronic acid (Sugahara *et al.*, 2016 ▸). The crystals first need to be checked to ensure that the crystallization solution is compatible with these viscous delivery media, the mixture produces a stable stream and the mixing process does not destroy any crystals. The primary advantage of HVE is much lower sample consumption due to a very low flow rate (0.01–3 µl min^−1^) compared with the GDVN.

**Figure 3:**
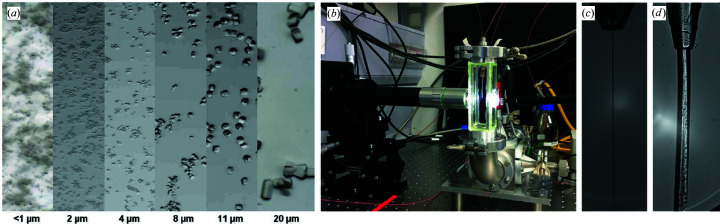
Sample optimization and injection test for SFX experiment. (*a*) Different sizes of lysozyme crystals, imaged with stereomicroscopes. The sizes of crystals need to be optimized for ideal sample injection. (*b*) An injection test chamber, equipped with an LED light source and a high-speed camera. With this setup, sample injection tests using (*c*) a GDVN and (*d*) HVE can be carried out.

The XBI facility offers the possibility to test different sample delivery methods in order to select the optimal one that maximizes the success of the experiments at the XFEL instruments. The injection test stations are designed to mimic the conditions at the instrument, using duplicates of the devices at the instruments or mock-ups when necessary. For more precise characterization of sample injection by a GDVN, an advanced setup with nanosecond laser illumination is planned, enabling high-jet-speed measurements. The current injection test station (Fig. 3 ▸) in the XBI laboratory consists of a small vacuum chamber, where injection properties of the sample, *e.g.* jet speed, length and diameter, can be measured. A high-speed camera and stroboscopic LED light source are used to record and characterize the flow properties of the sample and injector. HPLC pumps together with high-pressure helium gas connection and flowmeters for liquid and gas are used to adjust the jet speed and stability (Grünbein *et al.*, 2019 ▸). Testing of injection properties of the sample using the test chamber is needed to identify the ideal conditions for a stable jet in order to make optimal use of the available measurement time at the instruments. The jet diameter needs to be comparable to the X-ray beam size, as larger diameters may result in higher background. The diameter of the jet flow can be controlled by selection of the GDVN nozzle type in combination with liquid flow rate and sheath gas pressure.

### 2.2. Manufacturing nozzles for injectors   

There are many ways to manufacture nozzles for injectors. At the EuXFEL two methods are in place. One is the classical way of grinding the inner fused silica capillary on an abrasive plate and glueing it into a flame-polished outer borosilicate glass tube (DePonte *et al.*, 2009 ▸; Weierstall, 2014 ▸). In addition, 3D-printed nozzles are produced for the users. A 3D nanoprinter uses two-photon polymerization to print 3D microstructures with high lateral resolution (up to 170 nm), enabling also rapid prototyping and modifications of the nozzle. While the resulting hand-made GDVN nozzles vary, the printed nozzles provide a reliable and consistent jetting environment (Knoška *et al.*, 2020 ▸; Nazari *et al.*, 2020 ▸; Nelson *et al.*, 2016 ▸; Yefanov *et al.*, 2019 ▸). Two different HVEs (Botha *et al.*, 2015 ▸; Weierstall *et al.*, 2012 ▸) are also available for users with an option of 3D-printed nozzles. After the sample is prepared and fully characterized in the XBI laboratory, it is ready to be transferred to the SPB/SFX instrument via the sample elevator.

## 3. Imaging single particles in the gas phase   

Although experiments for structural characterization of biomolecules using XFELs have focused on SFX, significant effort has been devoted to the development of coherent diffractive imaging of single particles and biomolecules with the aim of obtaining structural information from samples without crystallization. The extremely short duration of XFEL pulses (of the order of tens of femtoseconds) provides an opportunity to outrun key damage processes during the exposure, resulting in snapshots virtually unperturbed by the probing X-rays (Chapman *et al.*, 2006 ▸, 2011 ▸, 2014 ▸; Neutze *et al.*, 2000 ▸; Oberthür, 2018 ▸; Wiedorn *et al.*, 2018 ▸).

Over recent years, SPI has overcome many technical challenges, resulting in the development of improved X-ray instrumentation, X-ray optics, and efficient computational phase retrieval and reconstruction algorithms (Bielecki *et al.*, 2020 ▸; Dashti *et al.*, 2020 ▸; Maia *et al.*, 2016 ▸). SPI experiments performed at XFELs have already produced proof-of-principle single-shot coherent diffraction images of viruses (Ekeberg *et al.*, 2016 ▸; Seibert *et al.*, 2011 ▸; Sobolev *et al.*, 2020 ▸), bacteriophages (Kassemeyer *et al.*, 2012 ▸), cell organelles (Hantke *et al.*, 2014 ▸) and cyano­bacteria that were alive at the time they were hit by the X-rays (van der Schot *et al.*, 2015 ▸). However, many challenges remain, such as for instance a need to increase the intensity and energy of the X-ray pulses together with high demands on detectors, which need to combine single-photon sensitivity with high dynamic range while also coping with the repetition rate of modern XFELs.

Concurrent with these ongoing efforts, sample preparation and delivery have been a major focus of research in the field of SPI. Similarly to cryo-electron microscopy, sample homogeneity is a key parameter for obtaining high-resolution reconstructions when averaging over a large number of single particles.

**Figure 4:**
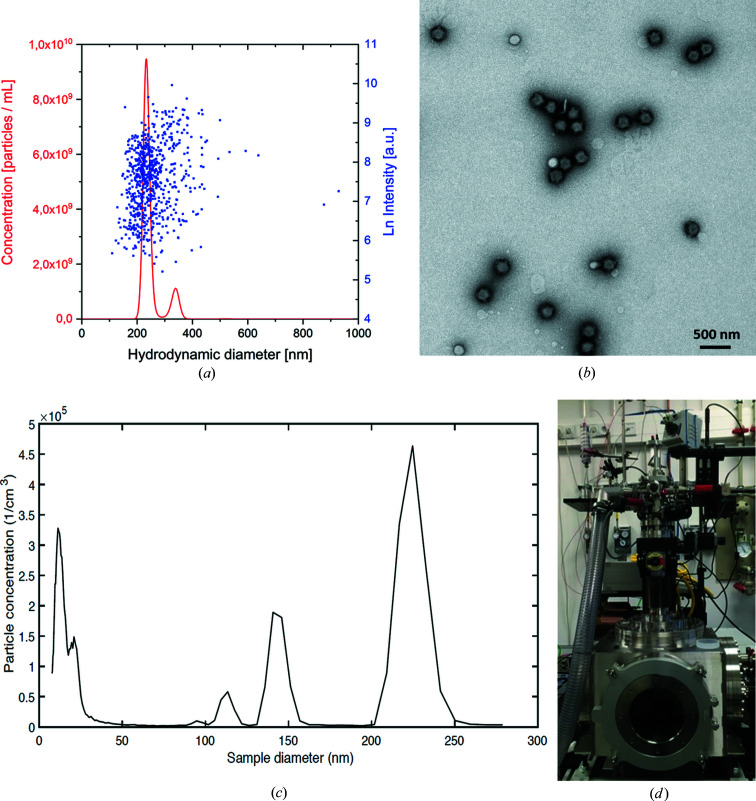
Example of sample characterization for single-particle imaging experiments using Melbourne virus particles (Lundholm *et al.*, 2018 ▸). (*a*) Particle size distribution and concentration determined by NTA based on individually tracked particles (blue) and integrated over all measured particles (red). (*b*) Negative stained TEM image of Melbourne virus particles. (*c*) Particle size distribution of aerosolized Melbourne virus particles measured by DMA. (*d*) Photographic image of the ‘Uppsala Injector’ (Hantke *et al.*, 2014 ▸) used for sample injection tests at the EuXFEL. Image (*d*) is courtesy of Johan Bielecki.

Fig. 4 ▸ shows examples of sample characterization methods available for single-particle imaging experiments at the XBI BioLab. The XBI laboratory provides a range of methods to test the integrity of the samples, which, to a large extent, are common for samples in SFX and SPI studies, including DLS, NTA and AUC. For aerosolized samples, a differential mobility analyser is available, which can provide information on the size distribution of particles in the gas phase (Kaufman *et al.*, 1996 ▸).

### 3.1. Aerosol sample injection   

Aerosol sample delivery lifts the requirement for sample support, which significantly reduces background scattering and allows for X-ray data collection from extremely small samples like single macromolecules at high rates (Hantke *et al.*, 2014 ▸; Ho *et al.*, 2020 ▸; Sobolev *et al.*, 2020 ▸). While aerosol sample delivery is an elegant approach with attractive advantages, it requires an aerosol injector that reaches high particle densities for achieving high hit ratios (*i.e.* fractions of XFEL pulses that hit at least one particle) and sufficient particle speed to prevent multiple exposures. The development of reliable techniques to create and characterize nanometre-sized droplets is important for numerous applications in physics, chemistry and biology, including studies in cluster physics, mass spectrometry, fuel injection, medications and cosmetics, and experiments where container-free sample handling is needed, *e.g.* in the study of isolated particles or macromolecules.

There are two ways of obtaining nanometre-sized droplets from dilute aqueous solutions. One is by fine-tuning a gas dynamic virtual nozzle (Mühlig *et al.*, 2019 ▸). A significant advantage of aerosolization with GDVNs is that the sample does not get charged. The other technique to reach even smaller droplets is electrospray ionization, coupled with charge reduction. The charge state of ions produced by electrospray ionization can be reduced in a controlled manner to yield predominantly singly charged ions (Scalf *et al.*, 2000 ▸).

Currently, the ‘Uppsala injector’ (Hantke *et al.*, 2014 ▸) is the most commonly used aerosol injector at XFELs, capable of delivering particles of 3–3000 nm diameter in a beam of about 10–15 µm diameter with very high number densities. The injector can be interfaced with a variety of aerosol sources and in the past has been used mainly with GDVNs. The injector was recently adapted to electrospray injection (Bielecki *et al.*, 2019 ▸). Electrospray injection is already a well established technique to aerosolize biological particles for mass spectrometry (Fenn *et al.*, 1989 ▸; Tito *et al.*, 2000 ▸; Uetrecht *et al.*, 2019 ▸) or differential mobility analysis (DMA) (Kaufman *et al.*, 1996 ▸). In order to minimize buffer deposition during the evaporation process, volatile buffers are usually used in SPI experiments (Bielecki *et al.*, 2019 ▸). DLS, NTA and DMA measurements should then be performed to optimize the buffer condition for sample injection and to validate the sample integrity (Fig. 4 ▸). An electrospray ‘Uppsala injector’ in combination with Rayleigh imaging (Hantke *et al.*, 2018 ▸) and DMA is available at the EuXFEL for sample injections tests. This allows optimizing sample quality, buffer conditions and injection parameters, and thus is an important step towards overcoming the sample delivery bottleneck in SPI of biological specimens at XFELs.

## 4. Alternative sample environments   

For certain applications, especially for rare or very expensive samples, alternative strategies for sample delivery may be necessary to reduce sample loss. The EuXFEL offers different ‘slow sample delivery methods’, primarily for SFX experiments, including high-viscosity extrusion (*e.g.* Roedig *et al.*, 2015 ▸; Nogly *et al.*, 2018 ▸). Drop-on-demand techniques through acoustic droplet ejection (Orville, 2017 ▸; Roessler *et al.*, 2016 ▸) or droplets deposited on a tape and delivered by a tape drive are in development (Fuller *et al.*, 2017 ▸). The XBI BioLab is available to test sample delivery with these injectors. A significant drawback of these ‘slow techniques’ is that they cannot utilize the entire megahertz pulse train of the European XFEL. Therefore, data collection time is prolonged, which is partially counteracted by a nearly 100% hit probability.

### 4.1. Experimental possibilities with fixed targets   

**Figure 5:**
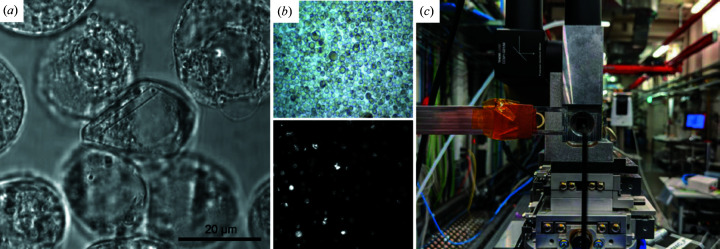
Example of samples and characterization for fixed-target measurements. (*a*) *In cellulo* grown protein crystal visualized by using a fluorescence microscope. (*b*) SONICC images (top: visible; bottom: SHG) of crystals grown in insect cells. (c) Fixed target installed on the Roadrunner in the downstream interaction region atmospheric pressure of the SPB/SFX instrument of the EuXFEL. Image (*b*) was taken by Robert Schönherr; image (*c*) is courtesy of Adam Round.

Fixed-target methods (Fig. 5 ▸) offer the possibility of dramatically reducing sample consumption and greatly improving data collection efficiency (Doak *et al.*, 2018 ▸; Hunter *et al.*, 2015 ▸; Mueller *et al.*, 2015 ▸). The relatively high background scattering can be reduced by using single-crystalline support materials equipped with micro-pores. In addition, micro-pores facilitate random orientation of the crystalline samples on the solid support. Combining fixed-target data acquisition with a humidity- and temperature-controlled environment in a helium atmosphere at atmospheric pressure allows not only fast sample exchange but also increased control of the measurement conditions compared with in-vacuum data collection (Roedig *et al.*, 2017 ▸). Fixed-target methods even provide the possibility of on-chip crystallization and *in situ* data collection, which minimizes mechanical stress due to crystal manipulation and the shear forces in high-pressure extrusion of the sample (Lieske *et al.*, 2019 ▸). These fixed crystals can then be introduced to the X-ray pulses by raster scanning the micro-patterned target, resulting in hit rates above 80%. By using a micro-patterned silicon chip in combination with the high-speed Roadrunner goniometer for sample delivery, complete data sets can be collected from a few micrograms of sample using less than 10 min of XFEL beam time. Other sample types like cells, bacteria or protein fibres can also be loaded onto a solid support, on which they tend to arrange themselves in a periodic fashion, according to the selected support material (Seuring *et al.*, 2018 ▸). Well suited for fixed-target sample delivery are protein crystals from ‘*in cellulo* crystallization’ (Koopmann *et al.*, 2012 ▸; Nass, Redecke *et al.*, 2020 ▸; Redecke *et al.*, 2013 ▸; Schönherr *et al.*, 2018 ▸). This recent non-conventional technique for protein crystallization is based on self-assembly of proteins in living cells (Brandariz-Nuñez *et al.*, 2010 ▸; Fan *et al.*, 1996 ▸; Gallat *et al.*, 2014 ▸). The large cell size (10–20 µm) is a limiting factor for liquid jet injection, but the cells can be either grown on or loaded onto solid supports for fixed-target measurements. *In cellulo* crystallization is supported in the XBI laboratory, combined with the possibility for data collection using the Roadrunner III (Roedig *et al.*, 2017 ▸) at the downstream interaction region of the SPB/SFX instrument of the European XFEL.

### 4.2. SPI with native mass spectrometry   

Work is in progress on using native mass spectrometry (MS) (Lorenzen & van Duijn, 2010 ▸) for sample selection and injection. If these attempts are successful, it will be possible to combine sample characterization with sample injection. The XBI laboratory has a native MS instrument that can be used to measure the molecular mass of large biomolecules up to the megadalton level, and determine the stoichiometry of protein complexes (Dülfer *et al.*, 2019 ▸). Electrospray injection is applied to aerosolize the particles and a time-of-flight analyser is used to determine mass-to-charge ratio. Such an arrangement allows scientists to select biomolecules according to their mass and conformation via ion mobility measurements and to partially orient molecules along their dipole axis (Uetrecht *et al.*, 2019 ▸). The EuXFEL is a partner in a project to integrate a native mass spectrometer (X-MS-I) into the SPB/SFX instrument for sample injection within the next few years.

## 5. Access to and availability of the XBI laboratory   

It is important that the unique opportunities offered by the European XFEL become available to a broad user community. The XBI BioLab and its support team help user groups in all aspects of sample preparation and characterization in connection to experiments at the EuXFEL. Sample preparation requires a significant investment of time and resources. When beam time is allocated, the facility initiates discussions with the users to support planning of sample preparation as well as testing and scoring of samples under the required conditions for experiments. Upon arrival on site, users are introduced to the laboratory and its equipment park. The local support staff provide guidance through all steps of sample preparation, characterization and testing. Many types of experiments can eventually be performed unsupervised, as users become more familiar with the XBI laboratory, its equipment and the best working practices. For complicated equipment, such as AFM or TEM instruments, a staff scientist conducts the measurements in collaboration with the users.

From the start of its operation, the XBI laboratory has been assisting users in performing exciting experiments. Three examples of publications acknowledging the use of the XBI laboratory are presented briefly below:

Example 1 is the first megahertz SFX experiment on membrane proteins (Gisriel *et al.*, 2019 ▸), which resulted in a 2.9 Å-resolution structure of the large membrane protein complex, photosystem I, containing 36 protein subunits and 381 cofactors. Obtaining large quantities of suitable crystals was challenging as crystal quality decreased during shipment to the EuXFEL. The use of the XBI laboratory was a key to the success of the experiment. All crystals used for X-ray measurements at the SPB/SFX instrument were freshly grown on site directly prior to the experiment, which ensured size homogeneity and avoided damage during transport.

Example 2 demonstrates that single-particle imaging can be performed using X-ray pulses at megahertz repetition rates (Sobolev *et al.*, 2020 ▸). This experiment demonstrated the possibility of reliable determination of particle size, scattering and background parameters, and proved the independence of X-ray pulses within a beam train. Samples of inorganic salts, Melbourne virus and mimivirus were prepared in the XBI laboratory and used at the SPB/SFX instrument for calibration and evaluation of the X-ray beam parameters.

Example 3 (Wiedorn *et al.*, 2018 ▸) presents two structures, a complex of CTX-M-14 β-lactamase with a covalently bound avibactam and lysozyme (as a test system), that were obtained from complete SFX data sets at 1.7 and 1.8 Å resolution, respectively. Microcrystals of hen egg white lysozyme and CTX-M-14 β-lactamase of up to 8 µm size were obtained in the XBI laboratory just before the experiment.

## 6. Summary   

The XBI facility offers the possibility for world class science and innovation to emerge from the European XFEL. The laboratory is versatile, has a broad profile, and can respond quickly to emerging scientific and societal challenges. The XBI BioLab is geared to support frontier research with projects of increasing complexity, sensitivity and scope.

## 7. Author contributions   

JH and VSL designed and initiated the XBI project. IK was the coordinator of the XBI User Consortium. KL supervised and guided the project from the construction phase to the completion of the bio lab and is the leader of the XBI BioLab. KL, HH, ER, RS, YG and JM wrote the first draft. The manuscript was improved by contributions from all authors. The final version of the manuscript was read and agreed to by all authors.
